# Robotic-assisted ureteroenteric reimplantation for ureteroenteric stricture after radical cystectomy: a systematic review and dual meta-analysis

**DOI:** 10.1007/s11701-025-02502-2

**Published:** 2025-07-06

**Authors:** Reham Ramadan, Mohamed Tharwat, Abdelwahab Hashem, Diaa-Eldin Taha

**Affiliations:** 1 Egyptian Ministry of Health, Dakahlia, Egypt; 2 Urology department, Faculty of Medicine, Delta University for science and Technology, Dakahlia, Egypt; 3 Urology department, Faculty of Medicine, Kafrelsheikh University, Kafr El-Sheikh, Egypt

**Keywords:** Robotic-assisted, ureteroenteric reimplantation, ureteroenteric stricture, radical cystectomy

## Abstract

**Background and Objective:**

Benign ureteroenteric stricture (UES) is a well-documented long-term complication that can occur after radical cystectomy with urinary diversion (UD). This meta-analysis evaluates the safety and feasibility of Robotic-assisted Ureteroenteric Reimplantation (RUER), with a focus on distinguishing outcomes between Open Ureteroenteric Reimplantation (OUER) and RUER.

**Methods:**

We performed an extensive search across multiple databases, including PubMed, Scopus, and Web of Science, to identify studies that assessed outcomes for RUER alone or in comparison to OUER. Relevant data were systematically extracted and recorded in an Excel sheet. Data analysis was performed using OpenMeta [Analyst] and Review Manager Software.

**Results:**

This review included 277 UES patients (244 RUER, 33 OUER) with 289 and 35 reimplantations, respectively. RUER had 91.7% success rate and a 7.1% stricture recurrence rate. Intraoperative complications, Clavien-Dindo ≥3 complications, conversion to open approach, and hospital readmission rates were 2.3%, 9.5%, 2.5%, and 7.8%, respectively. Compared to OUER, RUER showed similar success (RR =1.01, p=0.83) and stricture recurrence rates (RR = 0.89, p=0.72) but significantly lower intraoperative (RR =0.13, p=0.01), postoperative (RR = 0.53, p = 0.004), and Clavien-Dindo ≥3 complications (RR =0.27, p =0.01). RUER significantly reduced hospital stay (MD = -3.18 days, p = 0.0002) but showed no significant reduction in operative time (MD= -24.98 min, p=0.29).

**Conclusions:**

RUER offers comparable success to OUER with significantly lower complication rates and shorter hospital stays, making it a safe and feasible minimally invasive alternative for strictures ranging from 1 to 3 cm.

**Supplementary Information:**

The online version contains supplementary material available at 10.1007/s11701-025-02502-2.

## Introduction

Bladder cancer is among the top ten most prevalent cancers globally, with nearly 570,000 new cases and 210,000 cancer-related deaths reported in 2020 [1]. Radical cystectomy (RC) continues to be the primary treatment for patients with muscle-invasive bladder cancer (MIBC) and non-muscle-invasive bladder cancer (NMIBC) that is unresponsive to other therapies [2]. Urinary diversion involves anastomosis of the ureter to the bowel, most commonly the ileum. A significant and widely recognized long-term complication is benign ureteroenteric stricture (UES), occurring in approximately 4%–19% of patients who undergo radical cystectomy [3–5].

Ureteroenteric anastomotic strictures are typically categorized as either malignant or benign in etiology, with benign forms representing the vast majority. Benign ureteroenteric strictures are primarily caused by ischemia, often due to inadequate neovascularization of the distal ureter following its anastomosis with intestinal segments [6]. Additional risk factors—such as smoking, radiation, urinary tract infections, and urine leakage—can further impair healing and predispose to stricture formation [5–7].

The primary pathological mechanism involves periureteral fibrosis and scarring localized at the anastomotic site [8–10]. Inadequate surgical technique is a major contributor to ureteral ischemia; therefore, strict prevention strategies include meticulous preservation of the ureteral blood supply, gentle tissue handling, and minimization of electrocautery during dissection and reconstruction [11]. Notably, the incidence of strictures is higher on the left ureter than the right [12–14]. This is likely due to the increased mobilization and traction required when the left ureter is transposed beneath the sigmoid mesentery, which can impair vascular perfusion at the anastomotic site [11].

UES may cause irreversible loss of kidney function. However, its effective treatment remains complex [15, 16]. Endoscopic approach is commonly the first choice but have low long-term success rates and lacks long-term effectiveness [17–20]. Patients with rapid stricture development after urinary diversion, poor ipsilateral renal function, or left-sided UES have even lower success rates. Outcomes are also worse for strictures longer than 1 cm [18, 21].

Open ureteral reimplantation is highly effective but carries significant risks, especially in reoperative settings. In bladder cancer patients with multiple comorbidities, complications include anastomotic enterotomies, vascular injury, and a high postoperative complication rate within 90 days [18, 21, 22]. The need for a midline laparotomy further increases surgical morbidity [11, 21]. Reoperative procedures are additionally complicated by dense adhesions and peri-ureteral fibrosis, making ureter identification, urinary diversion, and precise delineation of UES margins challenging [11, 21, 23]. Because open surgery carries significant risks, many patients rely on long-term stent exchanges instead. But this approach has its own challenges, including potential complications, a decline in quality of life, and increased financial burden.

Over the last decade, as robotic platforms have become more widely adopted, a few centers have reported their initial outcomes on robotic-assisted UES reconstruction. Their goal has been to minimize the morbidity associated with traditional open surgery in these cases with success rates ranging from 91 to 96% [24–29]. However, data on its effectiveness remains limited.

We conducted this meta-analysis and systematic review to provide clearer insights by systematically reviewing existing evidence to better understand the safety, feasibility, and long-term benefits of Robotic-assisted Ureteroenteric Reimplantation (RUER). This meta-analysis estimates the effect of success rates, recurrence rates, intraoperative complications, Clavien-Dindo major complications, conversion to open surgery, and hospital readmission rates. Additionally, it compares Open Ureteroenteric Reimplantation (OUER) and RUER in terms of success rates, recurrence rates, intraoperative complications, postoperative complications, Clavien-Dindo major complications, operative time, and length of hospital stay.

## Materials and methods

### Study design

This meta-analysis was conducted strictly following the latest PRISMA guidelines and Cochrane Handbook [30, 31]. It is registered in PROSPERO under the record ID CRD42025649424.

We systematically searched Scopus, PubMed, and Web of Science using relevant keywords to identify studies, with the search updated up to June 2025. Supplementary File 1 provides the detailed search strategy.

### Inclusion criteria

We included studies involving patients with UES following radical cystectomy. The primary intervention was robotic-assisted ureteroenteric reimplantation (UER), with open UER or no control group as the comparison. Studies were eligible if they reported sufficient data on success rate and stricture recurrence, as well as other relevant outcomes such as intraoperative complications, major complications (Clavien-Dindo grade ≥ 3), length of hospital stay, operative time, hospital readmission, or conversion to an open approach. Studies were included if the reported data were compatible with extraction and analysis alongside other included studies. We considered original research, including randomized controlled trials (RCTs), cohort studies, non-randomized controlled trials, and case series, with no limitations on publication date to capture all relevant studies.

### Exclusion criteria

We excluded studies that did not provide sufficient data for extraction and analysis. Additionally, review articles, books, editorials, theses, and commentaries were not considered. Non-English language articles were also excluded. Furthermore, studies involving populations who underwent robotic or open surgery for ureteral reimplantation for causes other than UES following radical cystectomy were not included.

Using an Excel sheet, two authors initially screened the titles and abstracts of the collected studies. They then evaluated the full texts of the relevant studies to determine their eligibility. Any disagreements were resolved with input from a third author.

### Date extraction and study outcomes

Two independent authors gathered data from the relevant studies using a structured Excel file, which was divided into sections for study summaries, baseline information, and outcome measures. The summary sheet contained essential study details, including the study ID, location, involved site, duration, research methodology, participant number, open UER group, Robotic UER group, results, and follow-up duration post-reimplantation. The baseline table included various parameters such as the study ID, patient count, age, BMI, gender, reimplantation site, history of abdominal surgery, previous pelvic radiotherapy, prior neoadjuvant chemotherapy, past endoscopic procedures, cystectomy method, urinary diversion type, neobladder type, urinary diversion technique, preoperative creatinine levels, preoperative GFR, and stricture length.

The outcomes sheet contained key parameters, including sample size, operative time, intraoperative complications, postoperative complications within 30 days, Clavien-Dindo grade ≥ 3 complications, length of hospital stay, eGFR at the last follow-up, creatinine at the last follow-up, hospital readmission rates, number of cases converted to open surgery, overall success rate, and stricture recurrence. To determine the mean and standard deviations from the median and ranges for studies with continuous data, we applied specific statistical techniques [32]. Author consultations were used to address and resolve difference found throughout the data extraction process.

### Outcomes definition

***Success rate*** was confirmed using different methods across studies. Some studies assessed radiologic success through mercaptoacetyl-triglycine (MAG3) renal scans, where the absence of obstruction indicated a positive outcome, and loopograms, which evaluated the presence or resolution of reflux depending on the patient’s renal function [33]. Others confirmed success based on postoperative imaging, such as CT urograms or loopograms, to assess urinary tract patency [27].

*Stricture recurrence* was characterized by the occurrence of obstruction on a renogram, the necessity for renal drainage, the requirement for surgical intervention, or the need for nephrectomy [34].

### Risk of bias and quality of included studies assessment

The authors assessed study quality using the NIH tool for cohort studies, single-arm studies, and case series. [35]. We evaluated the quality of the evidence using the GRADE approach. This method takes into account factors like risk of bias, consistency of results, relevance to the research question, potential publication bias, and the precision of the findings [36]. All disagreements were settled by discussion.

### Data analysis

In order to conduct statistical analysis for the single-arm analysis, OpenMeta [Analyst] was used to calculate pooled estimates for categorical outcomes, presenting them with 95% confidence intervals. For the double-arm experiments, RevMan software version 5.4.1 was utilized. In this meta-analysis, mean differences (MD) and risk ratios (RR) were computed for continuous and categorical outcomes, respectively, and 95% CIs were given. Statistical significance was defined as a *p*-value of less than 0.05.

For single-arm analysis, a random-effects model was used from the outset to account for variability across studies. For double-arm analysis, a random-effects model was applied in cases of significant heterogeneity, which was assessed using the Chi-square Q test with a *p*-value threshold of 0.1. To test the robustness of the results, a leave-one-out sensitivity analysis was conducted.

## Results

### Data collection and study selection

**Fig. 1:**
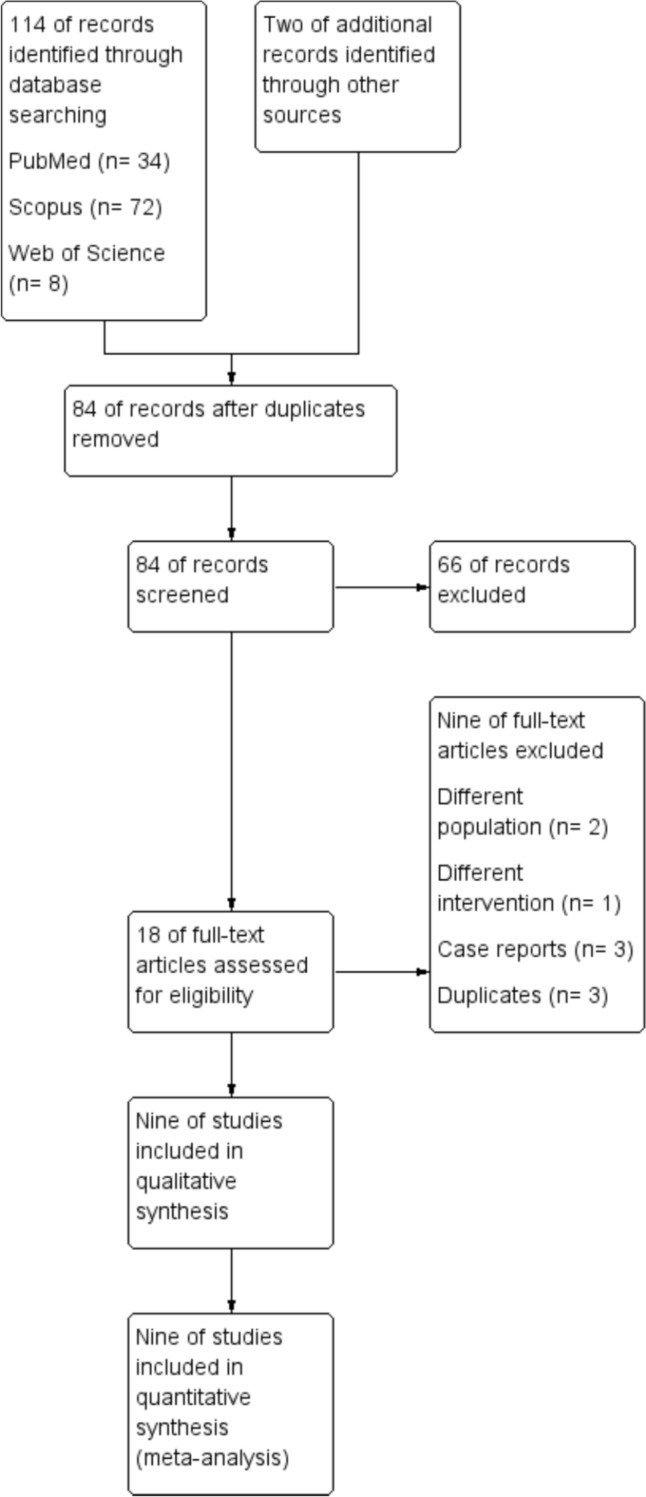
PRISMA Flow Diagram illustrating the study selection process, including records identified, screened, assessed for eligibility, and included in the final analysis

The electronic database search initially yielded 114 articles, along with two additional studies identified through manual search. Following the removal of duplicates, 84 articles proceeded to title and abstract screening, ultimately resulting in 18 articles being selected for full-text evaluation. We excluded three studies because they were case reports, and three others were duplicates. Additionally, two involved a different population, and one utilized a different intervention. Ultimately, only 9 studies were included in the synthesis. The study selection process is illustrated in Fig. 1.

This systematic review analyzed 277 patients diagnosed with UES following radical cystectomy; 244 underwent RUER, and 33 underwent OUER as a treatment intervention. Among them, a total of 289 ureteroenteric reimplantations were performed in the RUER group and 35 in the OUER group. A summary of the included studies is presented in Table 1.

**Table 1:** Summary of the included study

Study ID	Country, Site involved, and time of realization	Study design	Sample size (00 patients)	Open UER group	Robotic UER group	Result	Follow up ( months)
Ajami et al. [34]	Multi-center: Spain, USA, Sweden, between 2009 and 2021	Retrospective Cohort	80	All the open procedures were performed by the same center in order to compare the robotic approach with a standardized technique The open approach for ureteroenteric anastomosis is done through midline laparotomy, lysis of adhesions and the identification of the conduit or reservoir	The patient was positioned in a supine or lithotomy Trendelenburg position Four 8-mm robotic ports were placed transperitoneally, with optional assistant ports. Adhesiolysis was performed as needed, and ICG helped identify landmarks and strictures The ureter was dissected, transected, and reimplanted at a new site using absorbable sutures. Bricker anastomosis was used for unilateral UES, while Wallace or Bricker was used for bilateral cases A ureteral stent was placed transabdominally, with stent selection based on the type of urinary diversion	The median RUER operative time was 195 (175–269) min. No intraoperative complications or conversions to open approach were reported Twenty-three (37%) patients had postoperative complications (20 [32%] were minor and three [5%] major) The median length of hospital stay was 3 (1–6) d and readmissions were 5%. After a median follow-up of 19 (8–43) mo, 84% of cases were stricture free Lack of prior RT was the only variable associated with better stricture-free survival after RUER (hazard ratio 6.8, 95% confidence interval 1.10– 42.00, *p* = 0.037) The study limitations include its retrospective nature and the small number of patients	Median 37 (IQR 14.5–43.5)
	USA,between 2012 and 2018"	Retrospective Cohort	12	*	For the robotic approach, patients were positioned prone with port placement similar to robotic cystectomy. Adhesions were lysed, and ICG or guide-wire movement helped identify structures The ureter was transected, reimplanted using a non-refluxing technique, and stented retrograde through a catheter	One robotic case required conversion to open due to signifcant intestinal and peri-ureteral adhesions The median ages were 59 years in OR and 60 years in RR. Two patients in each group had failed previous endoscopic repair Median time from cystectomy to treatment of enteroanastamotic stricture was 13 months for OR and 10 months for RR (*p* = 0.25). Median estimated blood loss was 80 mL in both OR and RR (*p* = 1.0), median operative time was 260 min in OR and 255 min in RR (*p* = 0.13), and median hospital stay was 8 and 4 days, respectively (*p* = 0.06). There were two intra-operative and one post-operative complication in the OR group, one of whom required further surgical intervention, and no complications in the robotic cohort	2 years
Bearrick et al. [27]	USA, between January 2017 and October 2023	Retrospective Cohort	45	Open reconstruction was reserved for patients with lengthy left-sided strictures requiring tissue substitution. Patients with defect measuring > 4 cm on preoperative antegrade nephrostogram/loopogram or with stricture that appeared to the left of midline often needed tissue substitution in order to achieve a tension free repair	The patient is positioned in a modified flank position for antegrade ureteroscopy to assess the stricture. ICG or a secured ureteroscope aids visualization The robot is docked, and the ureter is carefully dissected and incised distally A stent is placed transabdominally using a guidewire, and the ureter is re-anastomosed to the conduit Tissue substitution is used for large gaps, and intravascular ICG assesses ureteral viability	UES repair was performed in 50 renal units a median of 13 months (interquartile range 7–30) from index surgery, and most often involved the left renal unit (34/50; 68%) Compared with robotic, open cases were significantly more likely to have undergone open cystectomy (100% vs 68%, *p* = 0.04), have longer strictures (median 4 vs 1 cm, *p* < 0.001), require tissue substitution (27% vs 3%, *p* = 0.04), and have lengthier postoperative hospitalization (5 vs 2 days, *p* < 0.001) There was no significant difference in total operative time (410 vs 322 min) or 30d major complications (18% vs 21%) At a follow-up of 13 months, per patient reconstructive success was 100% (11/11) for open and 97% (33/34) for robotic, respectively	Median18 (IQR 8–27)
Ghodoussipour et al. [26]	USA, Italy, between January 2010 and January 2019	Retrospective Cohort	46	*	Patients were positioned in Trendelenburg, with port placement based on prior surgeries. After lysing adhesions, the distal ureter and urinary diversion were mobilized The stricture was identified using ureteroscopy or dyes, excised, and the distal ureter was spatulated Direct anastomosis over a stent was performed with absorbable sutures, while Boari-like flaps were used for Padua pouch cases	Overall, 49 (84.5%) ureters underwent primary re-implantation, while 9 (15.5%) required Boari-like advancement flaps prior to re-implantation Median operative time was 190 min (range 45–540) with median estimated blood loss of 50 mL (range 25–2000) and median length of stay of 2 days (range 1–33, IQR 2–4) Seven (15.2%) patients experienced complications; 3 (6.5%) were low grade and 4 (8.7%) high grade. With median follow up of 18 months (range 1–51) the stricture recurrence rate was 8.6%	Median 18 (range 1–51)
Mahmood et al. [37]	"USA, between November 2005 and August 2023."	Retrospective Cohort	55	*	The patient was positioned in steep Trendelenburg, and robotic ports were placed based on the type of urinary diversion Adhesiolysis was often needed, and ICG or other methods helped identify the ureter and stricture The ureter was dissected carefully to preserve vascularity, transected at the stricture, and reimplanted using absorbable sutures A ureteral stent was optionally placed, though recent experience suggests it may not be necessary	The median time from RARC to UES was 4.4 (interquartile range 3.0e7.0) months, and the median time between UES and RUER was 5.2 (interquartile range 3.2e8.9) months The 3-year recurrence rate after RUER is about 29%. On multivariable analysis, longer hospital stay (hazard ratio 1.37, 95% confidence interval 1.16e1.61, *p*	Median 42.4 (IQR 16.9–56.6)
Rich et al. [25]	USA, Switzerland, Spain, Germany, Belgium, Sweden, Italy, the Netherlands, between January 2013 and September 2022"	Retrospective Cohort	31	*	Patients were positioned in Trendelenburg, with port placement adjusted based on stoma location Adhesiolysis was performed to prevent bowel injury and improve visualization The ureter was carefully dissected, avoiding excessive handling, and the stricture was identified and resected. Indocyanine green (ICG) and preoperative stenting aided in ureter identification The ureter was then spatulated and reimplanted using the Nesbit technique, with stents placed accordingly In bilateral strictures, the Wallace technique was used The anastomosis was completed with monofilament sutures, and leakage was checked by filling the conduit or neobladder with fluid	Median stricture length was 2.0 (1.0–3.25) cm, operative duration was141 (121–232) minutes, estimated blood loss was 100 (50–150) mL, and length of hospital stay was 5 (3–9) days One (3.2%) case was converted to open and one (3.2%) intraoperative complication occurred Seven (22.6%) patients experienced postoperative complications, including four (12.9%) Clavien–Dindo grade 3complications No Clavien–Dindo grade 4 or 5 complications occurred. Stricture recurrence occurred in 2 (6.5%)patients	Median 21 (IQR 9–43)
Sarychev et al. [28]	Switzerland, between May 2020 and March 2023	Retrospective case series	6	*	Standard Da Vinci surgical instruments were used The patient was positioned in a 30° Trendelenburg position, with port placement similar to that in robotic prostatectomy The pneumoperitoneum was established through a supraumbilical mini-laparotomy using the Hasson technique Adhesions around the neobladder were carefully freed. Subsequently, the affected ureter and the stricture were identified and localized This was achieved by intraluminal application of 10 mL of indocyanine green solution (2.5 mg/mL concentration) through the nephrostomy catheter The ureter was mobilized as needed. The ureteral stricture was identified and then fully excised To exclude any malignancy at the ureteral margin, a frozen section analysis was conducted. The ureter was then spatulated Reanastomosis between the ureter and neobladder was performed using a continuous 4–0 Stratafix suture A double-J ureteral catheter was inserted to secure the anastomosis, and the anastomosis was completed over this catheter	The mean operative time at the robotic console was 122 min, ranging from 80–160 min, and the mean blood loss was 42 mL, within a range of 50–100 mL Intraoperative frozen sections revealed no evidence of malignancy in all cases No postoperative complications exceeding Clavien-Dindo grade 3 were observed Two patients were treated for symptomatic urinary tract infections. The median length of stay in the hospital was 4 days, with a range of 2–7 days Median times for cystography with transurethral catheter removal and double-J catheter removal were 15 postoperative days (range: 12- 27) and 23 postoperative days (range: 17–37), respectively No recurrence of the condition was observed during a mean follow-up period of 23 months (range 6–40 months)	Median 23 (Range: 6–40 months)
Lee et al. [33]	USA, between 2013 and 2017	Retrospective case series	8	*	A retrospective review was conducted on patients undergoing robotic ureteroenteric reimplantation (RUER) with intraureteral and intraurinary diversion ICG between 2013 and 2017 Adhesiolysis and near-infrared fluorescence (NIRF) imaging guided ureteral dissection, identifying strictures and ensuring vascularized reimplantation sites Short-segment strictures were managed with Bricker or Wallace anastomoses, while long-segment cases used appendiceal interposition or transureteroureterostomy. Stents were placed transabdominally for support	The median operative time was 208 min (IQR 191–299), estimated blood loss was 125 ml (IQR 69–150), and length of stay was 6 days (IQR 1–8) Three of eight (37.5%) patients sufered a minor (Clavien ≤ 2), and 2/8 (25.0%) patients sufered a major (Clavien > 2) post-operative complication There were no complications related to ICG use At a median follow-up of 29 months (IQR 21–38), 8/10 (80.0%) ureteroenteric reimplantations were clinically and radiologically successful	Median 34 (IQR 21–38)
Tobis et al. [22]	USA	Retrospective case series	4	*	The da VinciSi Surgical System (Intuitive Surgical, Sunnyvale, CA) was used in all cases The port configuration is similar to robotic cystectomy, although the ports are placed in a more cephalad location on the abdominal wall The same port configuration was used for both right- and left-sided procedures Principal surgical techniques used include dissection of the colonic mesentery, careful peeling of the ureter off of the common iliac vessels, and mobilization of the ureter on either side of the sigmoid colon	Three of the 4 patients had undergone prior abdominal surgery in addition to their cystectomy All patients failed initial percutaneous and/or endourologic attempts to resolve their stricture. The ureteroileal strictures were successfully repaired robotically in all cases With mean follow-up of 16 months no major complications were encountered, and all patients remain free of stricture recurrence to date	Mean length of follow-up was 16 months

The average patient age in this review ranged from 61.33to 72 years. Preoperative eGFR levels varied between 33.85 and 68.43. A summary of the baseline demographics and clinical features is given in Table 2.

**Table 2:** Baseline demographic and clinical characteristics

`Study ID						Gender		Reimplantation side						
		Number of the patients	Number of ureteroenteric reimplantation	Age (years) mean&SD	BMI (mean&SD)	Male (n (%))	Female (n (%))	Right (n (%))	Left (n (%))	Bilateral (n (%))	Prior abdominal Surgery (n (%))	Prior pelvic radiotherapy (n (%))	Prior neoadjuvant chemotherapy (n (%))	Prior endoscopic procedure (n (%))
Ajami et al. [34]	Open UER	80	17	66.47 ± 5.66	24.64 ± 0.81	17(100)	0 (0)	3 (17.6)	9 (52.9)	5 (29.4)	8 (47.05)	0 (0)	2 (11.76)	3 (17.64)
	Robotic UER		65	66.29 ± 9.85	29.02 ± 6.56	54 (83)	11 (16.9)	23 (35.38)	35 (53.84)	7 (10.77)	30 (46.15)	5 (7.69)	26 (40)	28 (43.08)
	Open UER	5	7	61.33 ± 30.16	*	4 (80)	1 (20)	2(40)	1(20)	2 (40)	2 (40)	0 (0)	3(60)	2(40)
	Robotic UER	7	7	63.40 ± 26.63	*	6 (85.71)	1 (14.28)	3 (42.85(	4 (57.14)	0 (0)	1 (14.28)	1 (14.28)	2 (28.57)	2 (28.57)
Bearrick et al. [27]	Open UER	11	11	67.26 ± 8.48	27.77 ± 3.14	9 (81.81)	2 (18.18)	2 (18.18)	9 (81.81)	0 (0)	*	1 (9.09)	*	1 (9.09)
	Robotic UER	34	39	68 ± 7.73	29.46 ± 3.87	31 (91.17)	3 (8.82)	9 (26.47)	20 (58.82)	5 (14.7)	*	4 (11.76)	*	8 (23.52)
Ghodoussipour et al. [26]	Robotic UER	46	58	64.71 ± 9.04	27.84 ± 4.97	40 (86.95)	6 (13.04)	15 (32.60)	19 (41.30)	12 (26.08)	*	2 (4.34)	21 (45.65)	9 (19.56)
Mahmood et al. [37]	Robotic UER	45	55	66.70 ± 9.13	29 ± 6.09	49 (89.09)	6 (10.90)	18 (32.72)	37(67.27)	*	*	3 (5.45)	26 (47.27)	*
Rich et al. [25]	Robotic UER	31	43	66.11 ± 8.93	27.29 ± 6.22	29 (93.54)	2 (6.45)	6 (19.35)	13 (41.93)	12 (38.70)	*	*	6 (19.35)	*
Sarychev et al. [28]	Robotic UER	6	8	62 ± 4.75	*	*	*	2 (33.33)	2 (33.33)	2 (33.33)	*	*	*	*
Lee et al. [33]	Robotic UER	8	10	*	*	*	*	3 (37.5)	3 (37.5)	2 (25	*	1 (12.5)	*	5 (62.5)
Tobis et al. [22]	Robotic UER	4	4	72 ± 7.5	*	*	*	2 (50)	2 (50)	0 (0)	3 (75)	*	*	*

Study ID			Cystectomy approach			Urinary diversion			Type of neobladder			Urinary diversion approach		preoperative Creatinine (mg/dL)	preoperative GFR, (mL/min/m2)	Stricture length (cm)
			Open (n (%))	LAP (n (%))	Robotic (n (%))	Ileal conduit (n (%))	Neobladder (n (%))	Indiana pouch (n (%))	Studer (n (%))	Padovana (n (%))	Hautmann (n (%))	Intracorporeal (n (%))	Extracorporeal (n (%))	(mean&SD)	(mean&SD)	
Ajami et al. [34]	Open UER	80	10 (58.82)	3 (17.64)	4 (23.52)	9 (52.94)	8 (47.05)	*	2 (11.76)	3 (17.64)	3 (17.64)	4 (23.52)	0 (0)	1.3511 ± 0.29	55.08 ± 15.35	*
	Robotic UER		10 (15.38)	2 (3.07)	53 (81.53)	47 (72.30)	18 (27.69)	*	10 (15.38)	3 (4.61)	5 (7.69)	45 (69.23)	8 (12.30)	1.1335 ± 0.47	68.42 ± 31.69	*
	Open UER	5	*	*	1 (20)	*	*	*	*	*	*	*	*	*	44.92 ± 18.90	*
	Robotic UER	7	*	*	4 (57.14)	*	*	*	*	*	*	*	*	*	60.13 ± 15.52	*
Bearrick et al. [27]	Open UER	11	11 (100)	*	0 (0)	8 (72.72)	3 (27.27)	*	*	*	*	*	*	*	*	Median 4.0 (IQR 2.0–6.0)
	Robotic UER	34	23 (67.64)	*	11 (32.35)	23 (67.64)	11 (32.35)	*	*	*	*	*	*	*	*	Median1.0 (IQR 0.5–2.0)
Ghodoussipour et al. [26]	Robotic UER	46	1 (2.17)	*	45 (97.8)	18 (39.13)	26 (56.52)	1 (16.66)	11 (23.91)	15(32.60)	*	10 (21.73)	36 (78.26)	*	33.85 ± 18.61	Median1.5 (range 0.5–10)
Mahmood et al. [37]	Robotic UER	45	*	*	*	48 (87.27)	7 (12.72)	*	*	*	*	53 (96.36)	2 (3.63)	*	48.65 ± 22.83	*
Rich et al. [25]	Robotic UER	31	8 (25.80)	*	23 (74.19)	17 (54.83)	13 (41.93)	1 (3.22)	*	*	*	*	*	1.30 ± 0.34	*	Median 2 (IQR 1.0–3.25)
Sarychev et al. [28]	Robotic UER	6	2 (33.33)	*	4 (66.66)	2 (33.33)	4 (66.66)	*	*	*	*	*	*	*	*	*
Lee et al. [33]	Robotic UER	8	*	*	*	7 (87.5)	1 (12.5)	*	1 (12.5)	*	*	*	*	*	*	Median 2 (IQR 1–3)
Tobis et al. [22]	Robotic UER	4	*	*	*	*	*	*	*	*	*	*	*	*	*	*

### Quality assessment results

Most studies were rated as good quality, with only one classified as fair due to a lack of statistical methods clarification based on the NIH quality assessment tool [28, 35] (Table 3). The GRADE assessment showed low certainty of evidence for all outcomes, due to the observational nature of the included studies, along with concerns about imprecision or potential publication bias in some outcomes. Key outcomes—such as success rate, stricture recurrence, and complications—were rated as critical, while operative time and hospital readmission were considered important [36] (Table 4).

**Table 3:** NIH quality assessment tool for the included studies

Study ID	Item 1	Item 2	Item 3	Item 4	Item 5	Item 6	Item 7	Item 8	Item 9	Item 10	Item 11	Item 12	Item 13	Item 14	Score
Ajami et al. [34]	Yes	Yes	yes	yes	NR	yes	yes	Yes	yes	Yes	yes	NR	Yes	Yes	Good
	Yes	Yes	Yes	Yes	NR	yes	yes	yes	yes	yes	yes	NR	Yes	NR	Good
Bearrick et al. [27]	Yes	Yes	Yes	Yes	NR	yes	yes	yes	yes	yes	yes	NR	Yes	Yes	Good

Study ID	Item* 1	Item* 2	Item* 3	Item* 4	Item* 5	Item* 6	Item* 7	Item* 8	Item* 9	Item* 10	Item* 11	Item* 12	Score*
Ghodoussipour et al. [26]	Yes	Yes	Yes	Yes	Yes	Yes	Yes	NR	Yes	Yes	NR	NR	Good
Mahmood et al. [37]	Yes	Yes	Yes	Yes	Yes	Yes	Yes	NR	Yes	Yes	Yes	NR	Good
Rich et al. [25]	Yes	Yes	Yes	Yes	Yes	Yes	Yes	NR	Yes	Yes	NR	NR	Good

Study ID	Item^1	Item^ 2	Item^ 3	Item^ 4	Item^ 5	Item^ 6	Item^ 7	Item^ 8	Item^ 9	SCORE
Sarychev et al. [28]	Yes	Yes	NR	NR	Yes	Yes	Yes	NR	Yes	Fair
Lee et al. [33]	Yes	Yes	Yes	NR	Yes	Yes	Yes	Yes	Yes	Good
Tobis et al. [22]	Yes	Yes	Yes	NR	Yes	Yes	Yes	NR	Yes	Good

*CD, cannot determine; NA, not applicable; NR, not reported

For Cohort Studies:

Item 1: Research question; Item 2 and 3: Study population; Item 4: Groups recruited from the same population and uniform eligibility criteria; Item 5: Sample size justification; Item 6: Exposure assessed prior to outcome measurement; Item 7: Sufficient timeframe to see an effect; Item 8: Different levels of the exposure of interest; Item 9: Exposure measures and assessment; Item 10: Repeated exposure assessment; Item 11: Outcome measures; Item 12: Blinding of outcome assessors; Item 13: Follow-up rate; Item 14: Statistical analyses.

For Single-Arm Studies:

Item 1*: Research question; Item* 2: Study population; Item* 3: Representativeness of the study population; Item* 4: Enrollment criteria; Item* 5: Sample size justification; Item* 6: Consistency in intervention delivery; Item* 7: Outcome measures; Item* 8: Blinding of assessors; Item* 9: Follow-up rate; Item* 10: Statistical methods; Item* 11: Repeated outcome assessment; Item* 12: Group-level intervention analysis.

For Case Series Studies:

Item^ 1: Study question/objective; Item^ 2: Study population description; Item^ 3: Consecutive cases; Item^ 4: Comparability of subjects; Item^ 5: Intervention description; Item^ 6: Outcome measures; Item^ 7: Length of follow-up; Item^ 8: Statistical methods; Item^ 9: Results description.

**Table 4:** The certainty of evidence was assessed using the GRADE approach

Outcome	Study Design	No of Studies	No of Participants	Risk of Bias	Inconsistency	Indirectness	Imprecision	Publication Bias	Quality of Evidence	Effect Estimate	Importance
Success rate of RUER	Observational studies	9	289	Not Serious	Not Serious	Not Serious	Not Serious	Detected	● ● ○○ (Low	0.917 (95% CI: 0.870–0.964)	Critical
Stricture recurrence rate of RUER	Observational studies	9	289	Not Serious	Not Serious	Not Serious	Serious (-1)	Undetected	● ● ○ ○ (Low)	0.071 (95% CI: 0.032–0.110)	Critical
Intraoperative complications of RUER	Observational studies	6	187	Not Serious	Not Serious	Not Serious	Not Serious	Undetected	● ● ○ ○ (Low)	0.023 (95% CI: 0.002 to 0.044))	Critical
Clavien-Dindo (grade ≥ 3) complications of RUER	Observational studies	5	246	Not Serious	Not Serious	Not Serious	Serious (-1)	Undetected	● ● ○ ○ (Low)	0.095 (95% CI: 0.046–0.144)	Critical
Conversion to open approach	Observational studies	4	127	Not Serious	Not Serious	Not Serious	Not Serious	Undetected	● ● ○ ○ (Low)	was 0.025 (95% CI: 0.000–0.058)	Critical
Hospital readmission of RUER	Observational studies	2	120	Not Serious	Not Serious	Not Serious	Serious (-1)	Undetected*	● ● ○ ○ (Low)	0.078 (95% CI: 0.001–0.156)	Important

Success rate of RUER vs. OUER	Observational studies	3	146	Not Serious	Not Serious	Not Serious	Not Serious	Undetected*	● ● ○ ○ (Low)	RR = 1.01; 95% CI: 0.89–1.16)	Critical
Stricture recurrence rate of RUER vs. OUER		3	146	Not Serious	Not Serious	Not Serious	Serious (-1)	Undetected*	● ● ○ ○ (Low)	RR = 0.89; 95% CI: 0.49–2.82	Critical
Intraoperative complications of RUER vs. OUER		3	139	Not Serious	Not Serious	Not Serious	Not Serious	Undetected*	● ● ○ ○ (Low)	RR = 0.13; 95% CI: 0.03–0.65	Critical
Postoperative complications of RUER vs. OUER		3	139	Not Serious	Not Serious	Not Serious	Not Serious	Undetected*	● ● ○ ○ (Low)	RR = 0.53; 95% CI: 0.34–0.81	Critical
Clavien-Dindo (grade ≥ 3) complications of RUER vs. OUER		3	139	Not Serious	Not Serious	Not Serious	Not Serious	Undetected*	● ● ○ ○ (Low)	RR = 0.27; 95% CI: 0.09–0.77	Critical
Length of hospital stay of RUER vs. OUER		3	139	Not Serious	Not Serious	Not Serious	Not Serious	Undetected*	● ● ○ ○ (Low)	MD = -3.19; 95% CI: -4.89 to -1.49	Important
Operative time of RUER vs. OUER		3	57	Not Serious	Not Serious	Not Serious	Serious (-1)	Undetected*	● ● ○ ○ (Low)	MD = -24.98 (95% CI: -71.73 to -21.77	Important

**● ● ○ ○** indicates Low certainty of evidence

“*” Publication bias could not be formally assessed due to small number of studies; no evidence of selective reporting was observed

### Outcomes

#### Single arm analysis

**Fig. 2:**
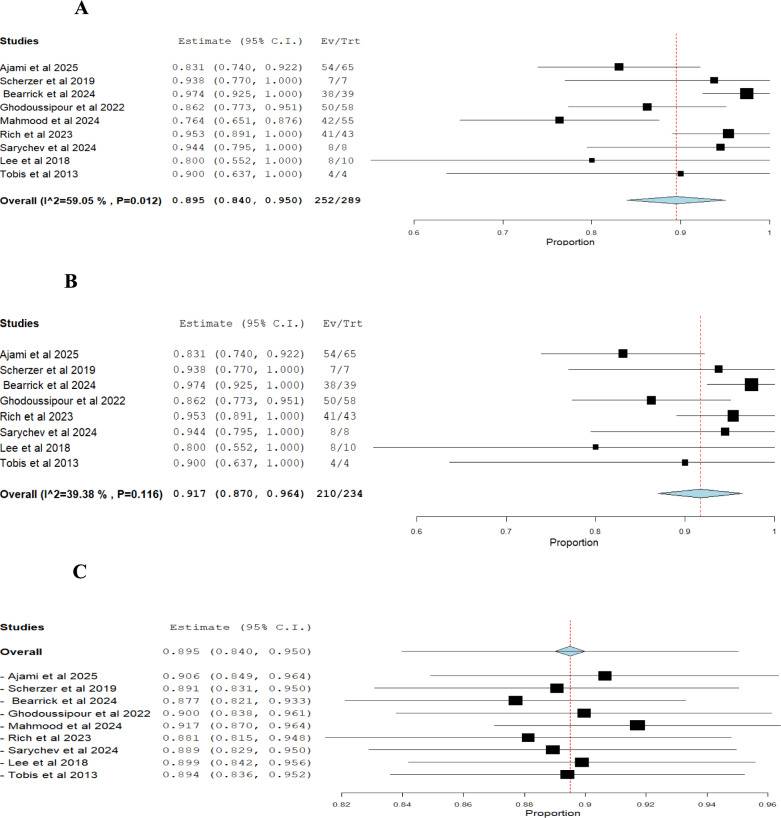
Single arm analysis of success rate of RUER

**Fig. 3:**
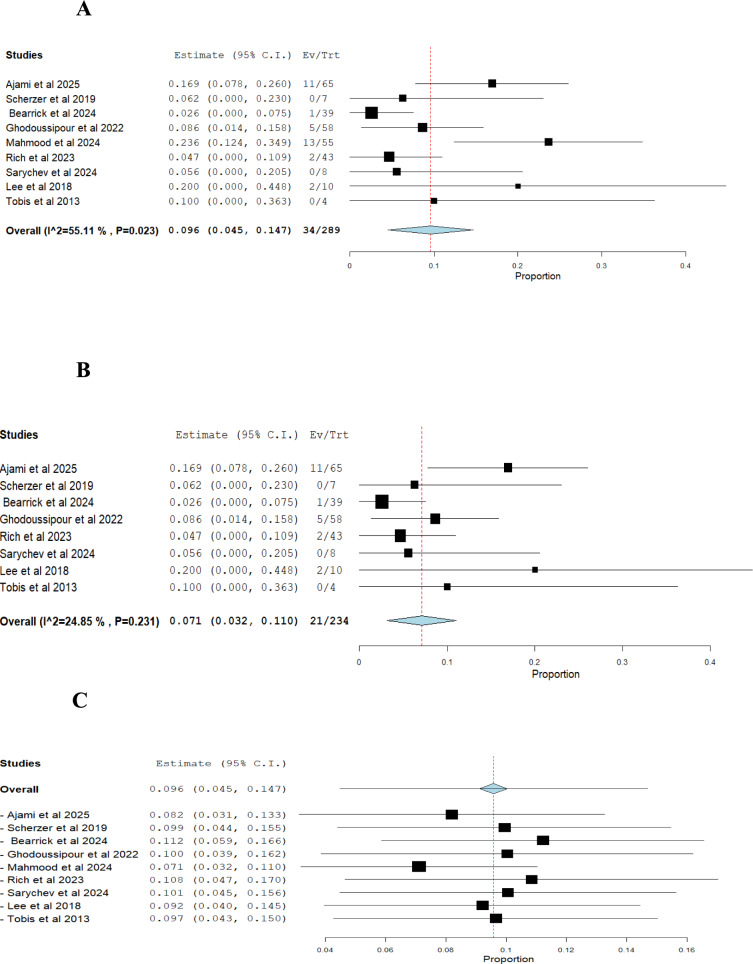
Single arm analysis of Stricture recurrence rate of RUER

**Fig. 4:**
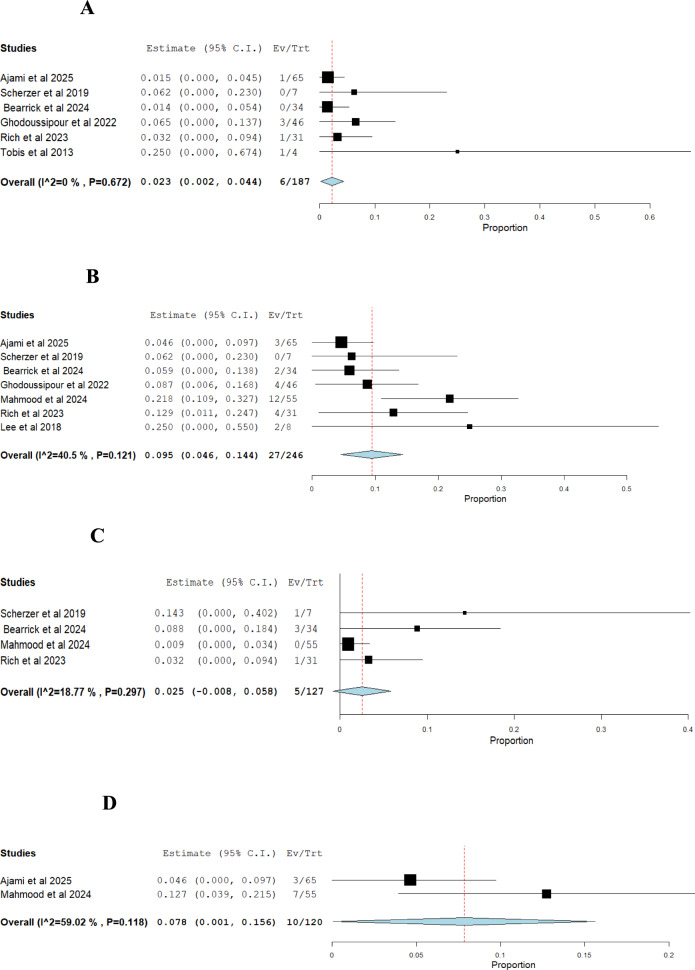
**A** Single arm analysis of intraoperative complication of RUER, **B** Single arm analysis of Clavien-Dindo (grade ≥ 3) complications of RUER, **C** Single arm analysis of Conversion to open approach, **D** Single arm analysis of Hospital readmission of RUER

i. *Success rate of RUER (based on number of ureteroenteric reimplantations)*: The pooled effect estimate was 0.895 (95% CI: 0.840–0.950), with moderate heterogeneity (I^2^ = 59.05%, *p* = 0.012) (Fig. 2A). Excluding Mahmood et al. (2024) adjusted the effect estimate to 0.917 (95% CI: 0.870–0.964), while heterogeneity decreased (I^2^ = 39.38%) and the *p*-value increased to 0.116 [37] (Figs. 2B, C).
ii. *Stricture recurrence rate of RUER (based on number of ureteroenteric reimplantations):* The pooled effect estimate was 0.096 (95% CI: 0.045–0.147), with moderate heterogeneity (I^2^ = 55.11%, *p* = 0.023) (Fig. 3A). Excluding Mahmood et al. (2024) adjusted the effect estimate to 0.071 (95% CI: 0.032–0.110), with heterogeneity decreasing (I^2^ = 24.85%) and the *p*-value increasing to 0.231 [37] (Figs. 3B, C).
iii. *Intraoperative complications of RUER:* The pooled estimate was 0.023 (95% CI: 0.002 to 0.044), with no statistically significant heterogeneity (I^2^ = 0%, *p* = 0.672) (Fig. 4A).
iv. *Clavien-Dindo (grade ≥ 3) complications of RUER:* The pooled estimate was 0.095 (95% CI: 0.046–0.144), with no statistically significant heterogeneity (I^2^ = 40.5%, *p* = 0.121) (Fig. 4B).
v. *Conversion to open approach:* The pooled estimate was 0.025 (95% CI: 0.000–0.058) with no statistically significant heterogeneity (I^2^ = 18.77%, *p* = 0.297) (Fig. 4C).
vi. *Hospital readmission of RUER:* The pooled estimate was 0.078 (95% CI: 0.001–0.156), with no statistically significant heterogeneity (I^2^ = 59.02%, *p* = 0.118) (Fig. 4D).

#### Douple arm analysis

**Fig. 5:**
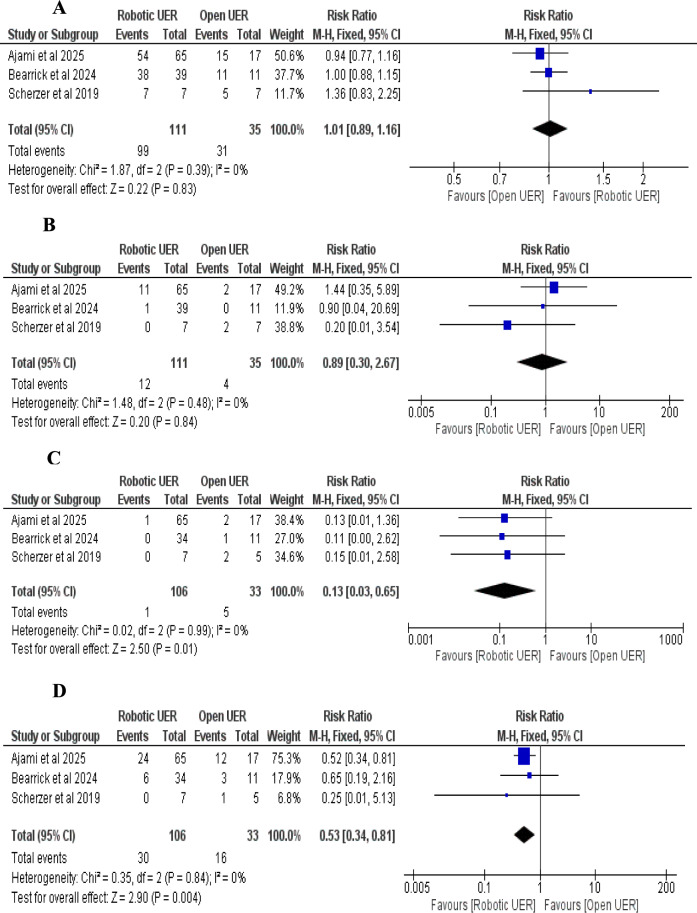
**A** Double-arm analysis of Success rate of RUER vs. OUER, **B** Double-arm analysis of Stricture recurrence rate of RUER vs. OUER, **C** Double-arm analysis of Intraoperative complications of RUER vs. OUER, **D** Double-arm analysis of Postoperative complications of RUER vs. OUER

**Fig. 6:**
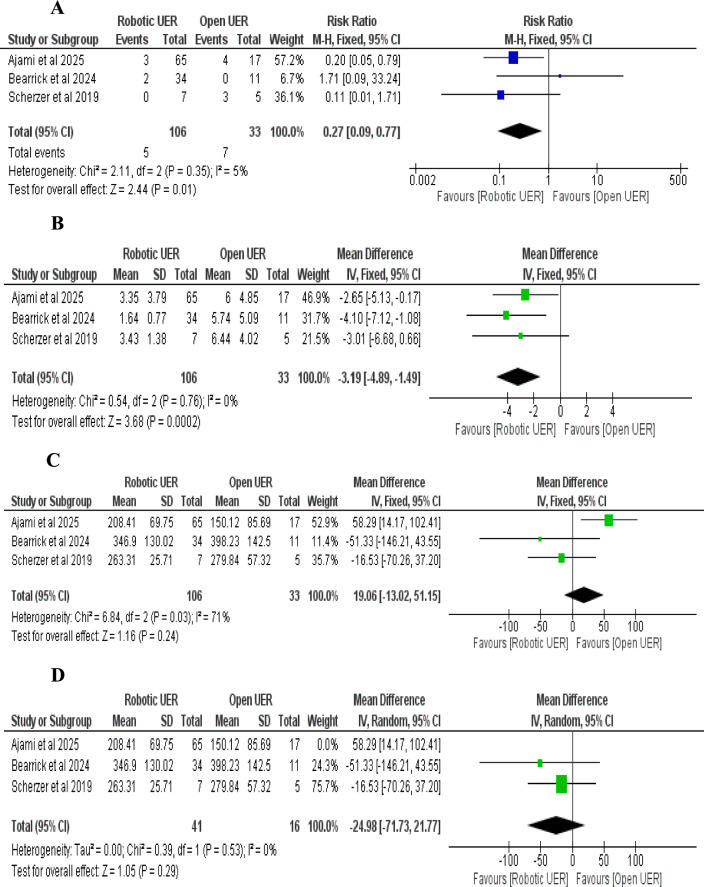
**A** Double-arm analysis of Clavien-Dindo (grade ≥ 3) complications of RUER vs. OUER, **B** Double-arm analysis of Length of hospital stay of RUER vs. OUER, **C,D** Double-arm analysis of Operative time of RUER vs. OUER

i. *Success rate of RUER vs. OUER (based on number of ureteroenteric reimplantations):* The pooled risk ratio (RR) showed no statistically significant difference between RUER and OUER (RR = 1.01; 95% CI: 0.89–1.16; *p* = 0.83). No heterogeneity was observed among the studies (I^2^ = 0%, *p* = 0.39) (Fig. 5A).
ii. *Stricture recurrence rate of RUER vs. OUER (based on number of ureteroenteric reimplantations):* The pooled risk ratio (RR) showed no statistically significant difference between the RUER and OUER groups (RR = 0.89; 95% CI: 0.49–2.82; *p* = 0.72). No heterogeneity was detected among the studies (I^2^ = 0%, *p* = 0.76) (Fig. 5B).
iii. *Intraoperative complications of RUER vs. OUER:* The pooled risk ratio (RR) significantly favored the RUER group over the OUER group (RR = 0.13; 95% CI: 0.03–0.65; *p* = 0.01). No heterogeneity was detected among the studies (I^2^ = 0%, *p* = 0.99) (Fig. 5C).
iv. *Postoperative complications of RUER vs. OUER:* The pooled risk ratio (RR) significantly favored the RUER group over the OUER group (RR = 0.53; 95% CI: 0.34–0.81; *p* = 0.004), with no statistically significant heterogeneity (I^2^ = 0%, *p* = 0.84) (Fig. 5D).
v. *Clavien-Dindo (grade ≥ 3) complications of RUER vs. OUER:* The pooled risk ratio (RR) significantly favored the RUER group over the OUER group (RR = 0.27; 95% CI: 0.09–0.77; *p* = 0.01), with no statistically significant heterogeneity (I^2^ = 5%, *p* = 0.35) (Fig. 6A).
vi. *Length of hospital stay of RUER vs. OUER:* The pooled mean difference (MD) significantly favored the RUER group over the OUER group (MD = -3.19 days; 95% CI: -4.89 to -1.49; *p* = 0.0002). The studies showed no signs of heterogeneity (I.^2^ = 0%, *p* = 0.76) (Fig. 6B)
vii. *Operative time of RUER vs. OUER:* The pooled mean difference (MD) was 19.06 min (95% CI: -13.02 to 51.15; P = 0.24). It showed no significant difference between groups, with moderate heterogeneity (I^2^ = 71%, *p* = 0.03) (Fig. 6C). Excluding Ajami et al. (2025) reduced heterogeneity to 0% (*p* = 0.53) and adjusted the effect estimate to -24.98 min (95% CI: -71.73 to -21.77; P = 0.29) [34] (Figs. 6D).

## Discussion

We analyzed data from 277 patients diagnosed with UES after radical cystectomy, with 244 undergoing RUER and 33 undergoing OUER, resulting in 289 and 35 ureteroenteric reimplantations, respectively. The Single arm-analysis showed that RUER had a high success rate (91.7%) with a low stricture recurrence rate (7.1%). Intraoperative complications were rare (2.3%), and the rate of severe Clavien-Dindo grade ≥ 3 complications was low (9.5%). Conversion to open surgery occurred in only 2.5% of cases, and the hospital readmission rate was 7.8%. In the double-arm analysis, RUER demonstrated no significant difference of success and stricture recurrence rates to OUER. However, RUER associated with significantly lower intraoperative and postoperative complications, including severe Clavien-Dindo grade ≥ 3 events. Patients undergoing RUER had a shorter hospital stay, while operative time showed variability.

It may be challenging to manage ureteroenteric anastomotic strictures following a radical cystectomy. Since there are no guidelines, the majority of specific suggestions are based on the opinions of experts [13, 18, 38, 39]. Reconstructive surgery, particularly after major procedures, has evolved from primarily open techniques to more minimally invasive approaches. Robot-assisted surgery has played a crucial role in this shift by overcoming some of the limitations of standard laparoscopic techniques. Advances in robotic technology, along with improved surgical training, have expanded the complexity of cases that can be managed robotically. This approach may help reduce complications, especially in older patients with multiple health conditions.

Endoscopic treatment is typically the first-line approach, often performed using an antegrade technique due to the challenges of retrograde access. However, these methods have poor long-term success rates. The long-term outcomes of minimally invasive intervention for UES were examined by Schondorf et al. At a median follow-up of 29 months, endourological interventions had an overall success rate of 26%, with modality-specific success rates of 33% for both endoureterotomy and Ho:YAG laser endoureterotomy, and 25% for balloon dilation [38]. The endoscopic success rate was 50% for strictures that were 1 cm or less, and it was close to 8% for strictures that were more than 1 cm. Poor long-term success rates of endoscopic treatments have been verified by earlier research [13, 40].

Due to the limited long-term success of endoscopic treatment, open revision remains the preferred standard for managing UES [41]. In a study of 151 patients, Packiam et al. assessed open UES repair, reporting a success rate of 93.4%. The most common intraoperative complication was deserosalization (8.1%), followed by enterotomies requiring bowel anastomosis (3.2%) and significant vascular injuries (2.4%). In contrast, our meta-analysis found that RUER had a success rate of 91.7% with a much lower intraoperative complication rate of 2.3%. Additionally, Packiam et al. reported that 48% of patients experienced postoperative complications within 90 days, with 12.1% classified as high-grade. Our meta-analysis showed a lower rate of severe complications (9.5%). Packiam et al. reported a 30-day readmission rate of 9.7% and a stricture recurrence rate of 6.6% for OUER, while our meta-analysis found a recurrence rate of 7.1% for RUER and a readmission rate of 7.8% [21]

According to Carrion et al., prior abdominal or pelvic radiation therapy (RT) was a key predictor of RUER success. Patients without prior RT had a higher success rate (86.2%) compared to those with RT (60%). Multivariate analysis confirmed that the absence of RT was the only significant factor associated with better stricture-free survival (hazard ratio 6.80) [24]. Prior studies have identified several risk factors for ureteroenteric stricture formation after cystectomy, including patient-related factors (such as smoking history and prior radiation), surgical factors (such as continuous suturing or lack of postoperative stenting), and postoperative complications (such as urinary tract infections and urine leakage) [6, 7, 42]. Nevertheless, the predictors of stricture recurrence after ureteral reimplantation are not well established, with stricture length greater than 1 cm being the only identified one [18]. Packiam et al. found a weak association between stricture recurrence and factors such as a history of smoking, urinary leakage, and previous radiation therapy. While left-sided strictures are generally linked to a higher recurrence risk and poorer outcomes after endoscopic treatment, they had little impact on success rates following open ureteral reimplantation [21].

Bearick et al. noted that the best outcomes for RUER are seen in short, focal strictures (< 2 cm), while longer, left-sided strictures lateral to the midline often require proximal ureter and sigmoid colon mobilization, sometimes necessitating tissue substitution. The choice between robotic and open surgery depends on the surgeon’s expertise, with complex cases potentially favoring an open approach [27]. Meanwhile, Ghodoussipour et al. reported a 91.4% success rate for RUER, with a median stricture length of 1.5 cm (range: 0.5–10 cm), demonstrating its effectiveness even in longer strictures [26]. Furthermore, our meta-analysis found an overall success rate of 91.7% in RUER cases, with a pooled median stricture length of 1.75 cm (IQR: 1.0–3.0) [25–27, 33].

Recent evidence suggests that adjunctive use of ICG in robotic urologic procedures may facilitate intraoperative identification of strictures and improve vascular assessment [43, 44]. Tuderti et al. reported favorable outcomes using transnephrostomic ICG during robotic ureteral reimplantation in 10 patients post-cystectomy, with only one recurrence and no renal function decline after 19 months of follow-up [45]. Similarly, Ahmadi et al. evaluated 179 patients undergoing robotic cystectomy with intracorporeal diversion, finding a 0% stricture rate in the ICG group versus 10.6% per-patient and 6.6% per-ureter in the non-ICG group (*p* = 0.020 and *p* = 0.013, respectively). They concluded that using ICG to assess distal ureteric vascularity during robotic cystectomy with intracorporeal diversion may reduce the risk of uretero-enteric strictures [46].

In the setting of ureteroenteric strictures (UES), as a result of intraperitoneal adhesions and periureteral fibrosis, identifying the ureters and urinary diversion in patients with UES can be challenging [37]. RUER with intraureteral injection of ICG has been reported to lower the risk of bowel and vascular damage and provide rapid and accurate ureter identification [33, 45]. Just 17% of Carrion et al.'s patients received intraureteral ICG, but they have not report any instances of intraoperative complications or conversion to open surgery [24]. Robot-assisted surgery has limitations, including challenges in accessing surgically altered abdominal areas and the lack of haptic feedback. However, Dangle and Abaza proposed that robot-assisted radical cystectomy (RARC) may lead to fewer intraperitoneal adhesions compared to open surgery. This could make ureteroenteric reimplantation easier for these patients [23].

One of the key strengths of this systematic review is its adherence to the latest reporting guidelines for systematic reviews and meta-analyses. It also provides a detailed analysis of existing studies. Furthermore, the study provides a detailed range of outcomes. We successfully addressed heterogeneity among the included studies. Finally, we aim to bridge a crucial gap in the existing literature.

However, this review has some limitations, such as the absence of randomized controlled trials (RCTs) on this approach. We also included studies with varying designs, and the number of head-to-head comparative studies between robotic and open ureteroenteric reimplantation was limited. Although heterogeneity across studies was generally low, this should be interpreted with caution given the small number of included comparative studies. Additionally, the small sample size restricted the strength of our conclusions. Larger, well-designed RCTs and prospective comparative studies with longer follow-up are essential to validate these findings and provide more definitive evidence.

## Conclusion

Robot-assisted ureteral reimplantation (RUER) is a safe and effective approach for managing ureteroenteric strictures after radical cystectomy. It offers comparable success to open ureteral reimplantation (OUER) while significantly reducing complication rates and hospital stays. With careful patient selection, particularly for strictures ranging from 1 to 3 cm, RUER can serve as a minimally invasive alternative to open surgery. It can minimize morbidity and potentially reduce the need for long-term stenting or renal deterioration. Further well-designed comparative studies are needed to improve the certainty of current evidence.

## Supplementary Information

Below is the link to the electronic supplementary material.

Supplementary file1 (DOCX 21 KB)

## Data Availability

No datasets were generated or analysed during the current study.
