# Supplementary material for: Robotic-assisted ureteroenteric reimplantation for ureteroenteric stricture after radical cystectomy: a systematic review and dual meta-analysis
Source: J Robot Surg. 2025 Jul 6;19(1):356. doi: 10.1007/s11701-025-02502-2 (PMC12229966; doi:10.1007/s11701-025-02502-2)
Supplement: Supplementary file 1 — Supplementary file1 (DOCX 21 KB) [file 11701_2025_2502_MOESM1_ESM.docx]

***Search strategy*:**

***PUBMED:***

(“Radical cystectomy” OR “radical cystectomies” OR “cystectomy” OR “Bladder removal” OR “RC” OR “Cystectomies” OR “Cystectomy, Radical” OR “Cystectomy, Radical” OR “Cystectomy, Radical” OR "Cystectomy"[Mesh]) OR “Ureteroenteric Strictures” OR “ureteroenteric anastomotic strictures” OR “UAS” OR “Uretero-enteric Strictures” OR “UES”) AND (“Open ureteroenteric reimplantation” OR “OUER” OR “Open uretero-enteric reimplantation” OR “open reimplantation of the ureter” OR “Robot-assisted ureteroenteric reimplantation” OR “RUER” OR “robotic ureteral reimplant” OR “robot-assisted repair of uretero-enteric reimplantation” OR “robotic ureteral reimplantation” OR “robot assisted uretero-enteric reimplantation”)

*Date of search: January 2025*

*Search results: 34*

*No limitations applied*

***SCOPUS***

ALL ( (“Radical cystectomy” OR “radical cystectomies” OR “cystectomy” OR “Bladder removal” OR “RC” OR “Cystectomies” OR “Cystectomy, Radical” OR “Cystectomy, Radical” OR “Cystectomy, Radical” OR “Ureteroenteric Strictures” OR “ureteroenteric anastomotic strictures” OR “UAS” OR “Uretero-enteric Strictures” OR “UES”) AND (“Open ureteroenteric reimplantation” OR “OUER” OR “Open uretero-enteric reimplantation” OR “open reimplantation of the ureter” OR “Robot-assisted ureteroenteric reimplantation” OR “RUER” OR “robotic ureteral reimplant” OR “robot-assisted repair of uretero-enteric reimplantation” OR “robotic ureteral reimplantation” OR “robot assisted uretero-enteric reimplantation” ) )

*Date of search: January 2025*

*Search results: 72*

*No limitations applied*

***WEB OF SCIENCE***

ALL ( (“Radical cystectomy” OR “radical cystectomies” OR “cystectomy” OR “Bladder removal” OR “RC” OR “Cystectomies” OR “Cystectomy, Radical” OR “Cystectomy, Radical” OR “Cystectomy, Radical” OR “Ureteroenteric Strictures” OR “ureteroenteric anastomotic strictures” OR “UAS” OR “Uretero-enteric Strictures” OR “UES”) AND (“Open ureteroenteric reimplantation” OR “OUER” OR “Open uretero-enteric reimplantation” OR “open reimplantation of the ureter” OR “Robot-assisted ureteroenteric reimplantation” OR “RUER” OR “robotic ureteral reimplant” OR “robot-assisted repair of uretero-enteric reimplantation” OR “robotic ureteral reimplantation” OR “robot assisted uretero-enteric reimplantation”) )

*Date of search: January 2025*

*Search results: 8*

*No limitations applied*
